# Supplementary material for: Myo-inositol versus D-chiro-inositol in murine in vitro follicular development: An experimental study relevant to polycystic ovary syndrome
Source: PLoS One. 2026 Mar 31;21(3):e0346159. doi: 10.1371/journal.pone.0346159 (PMC13037987; doi:10.1371/journal.pone.0346159)
Supplement: Attachment — Submitted filename: Response_to_Reviewers.docx [file pone.0346159.s002.docx]

# Response to Reviewers

Response to Reviewer #1

Major Comments

1. Novelty and Significance

While the topic is relevant, the novelty should be better emphasized in the introduction and discussion. Many prior studies have reported MI/DCI in PCOS, but this work’s unique value (direct follicle culture model) should be more clearly highlighted.

⇒We appreciate this valuable suggestion. We have revised both the Introduction and Discussion to emphasize that the novelty of our study lies in the use of a direct murine single-follicle culture system.

**Modification:**

Added in *Introduction* (lines **75-83**)

“Although MI and DCI have shown efficacy in treating PCOS, most studies have focused on their metabolic effects, and the direct impact of MI/DCI on follicular development remains poorly understood. In particular, the influence of altered MI/DCI ratios in PCOS on follicular development has not been fully investigated, and animal model studies are limited. Therefore, this study aimed to examine the direct effects of MI and DCI on secondary follicle development in mice to enhance understanding of PCOS pathophysiology and inform therapeutic strategies. To isolate these stage-specific actions from systemic factors, we employed a single-follicle culture system based on established methods for multistage in vitro follicular development”

2. Statistical Power and Sample Size

Follicles were obtained from 8 mice in total (n=12 follicles per group). This is a relatively small sample size, and variability in follicle culture is usually high. The authors should justify whether this number provides sufficient statistical power.

**⇒**Thank you for this important point. We agree that follicle culture systems inherently involve variability. In the revised *Methods*, we clarified that our sample size (n=12 follicles per group, from 16 mice) was consistent with prior in vitro follicle culture studies that reported comparable statistical robustness. Moreover, data were tested for normality (Shapiro–Wilk test) and analyzed one-way ANOVA followed by Student–Newman–Keuls post hoc test to ensure reliable intergroup comparison.

**Modification:**

Added clarification in *Sample size and power* section (lines **162-172**)

“Sample size and power

To adhere to the 3Rs principle, particularly the principle of reduction, a single follicle culture system was adopted. This allowed multiple experimental units to be obtained from each animal. In total, follicles were collected from 16 female ICR mice. To avoid allocation bias, follicles from both ovaries were used, with 12 follicles randomly assigned to each group.

This design aligns with established single follicle culture studies (our previous work [17]), where 3–5 animals per experiment were sufficient to detect biologically meaningful differences.

Considering the observed range of follicle diameter variation (standard deviation approximately 10–15 μm), this sample size was deemed adequate to detect a moderate effect using one-way ANOVA (α=0.05).”

**We acknowledge that the Student–Newman–Keuls test is less conservative than Bonferroni; however, it was chosen to balance type I error control with moderate-effect detection in small-sample designs.**

3. Interpretation of Results

The authors conclude that DCI promotes the transition of secondary to early antral follicles, but the effect was transient (lost by day 10). This should be discussed more critically - is this effect biologically meaningful or simply a short-term in vitro artifact?

The discussion suggests a possible involvement of the PI3K/AKT signaling pathway, but no experiments were conducted to test this hypothesis. The speculative nature of this statement should be acknowledged more clearly.

**⇒**We appreciate this thoughtful comment. We have revised the *Discussion* to clarify that the DCI-mediated acceleration appears to be **stage-specific and transient**, likely reflecting the known **growth ceiling (~300 μm)** of single-follicle systems. We now explicitly acknowledge that **an in vitro artifact cannot be excluded**, and that further long-term or co-culture models are needed to confirm biological relevance.

**Modification:**

Revised paragraph in *Discussion* (lines **274-283**)

“Follicles cultured without DCI, although initially delayed, eventually reached similar developmental stages as those exposed to DCI. Thus, lowering the MI/DCI ratio by DCI supplementation did not reproduce the late-stage arrest typically observed in PCOS. However, our findings provide stage-specific, in vitro mechanistic evidence for DCI’s role in promoting the transition from secondary to early antral follicles. This discrepancy between early- and late-stage effects is likely due to the limitations of the single-follicle culture system, in which follicle growth appears to plateau at approximately 300 μm. Future experiments should evaluate MI/DCI effects under lower FSH concentrations to minimize gonadotropin influence, test a broader range of MI/DCI doses, and validate findings in co-culture systems that more closely replicate the ovarian microenvironment.”

4. MI Data Interpretation

MI showed no significant effect. The authors attribute this to a high FSH concentration and a low MI dose; however, they should expand on why the chosen doses were used and whether pilot experiments with higher MI levels were considered.

**⇒** We expanded the *Discussion* to explain that MI concentrations (20–50 μM) were selected to mirror physiological levels in follicular fluid of women with and without PCOS. These concentrations are considerably lower than those typically used in in vitro maturation (IVM) systems. We now explicitly acknowledge that higher MI levels were not tested and identify this as a limitation.

**Modification:**

Revised section in *Discussion* (lines **294-299**):

“MI, known as a second messenger of FSH, did not significantly affect follicle growth or E2 levels in this study. The high FSH concentration used (~66–67 mIU/mL) may have masked subtle MI-dependent effects. Additionally, the MI concentrations applied (20–50 μM) reflected physiological or PCOS follicular-fluid levels, whereas many in vitro maturation studies use millimolar concentrations [20]. Future pilot studies using higher MI doses, under controlled osmolarity conditions are warranted to further clarify these effects.”

5. Clinical Relevance

The link to PCOS patients is somewhat overstated. Since this is a murine follicle culture system, the limitations for extrapolating to humans should be described more carefully.

⇒ We agree that extrapolation to human PCOS must be made more cautiously. The revised Discussion now explicitly states that findings are mechanistic and stage-specific, not directly clinical, and briefly discusses interspecies differences in ovulatory pattern and follicular morphology.

**Modification:**

Revised paragraph in *Discussion* (lines **300-307**):

“The single-follicle murine model has inherent constraints regarding direct clinical extrapolation. Mice are polyovulatory and exhibit multifollicular ovarian morphology, while humans are monovulatory; therefore, the follicular dynamics of mice do not fully replicate those of human ovaries. Additionally, murine follicles have shorter growth periods and differing endocrine sensitivity compared with human follicles. Accordingly, these results should be interpreted as mechanistic, stage-specific in vitro findings rather than clinical conclusions. Further studies using human or non-human primate follicles would help elucidate these processes.”

More Comments

1. Language and Style

English is understandable but requires polishing for clarity and conciseness. Example:

Abstract:

In lines 20-23, “This study investigated the effects of myo-inositol and d-chiro-inositol on the development of murine ovarian follicles in vitro, and their potential efficacy in patients with polycystic ovary syndrome.”

- “their potential efficacy in patients” is misleading, because the authors did not test patients, only mice.

- “myo-inositol and d-chiro-inositol”, chemical names should be italicized consistently or abbreviated after first use.

⇒Thank you for your suggestion. We have revised the relevant section as follows:

This study investigated the effects of myo-inositol (MI) and D-chiro-inositol (DCI)on the development of murine ovarian follicles in vitro. (Lines 22-24)

Lines 23-25, “d-chiro-inositol treatment exhibited significantly larger diameters compared to the control group on Day 6 (D=275.20 ± 12.54 μm; p=0.037) and Day 8 (D=277.47 11.47 μm; p=0.048), but this difference was no longer observed by Day 10.”

“exhibited significantly larger diameters”, awkward phrasing; better: “resulted in significantly larger follicle diameters.”

“277.47 11.47 μm” → missing ± sign.

“but this difference was no longer observed” → wordy.

The correct sentence could be: DCI treatment resulted in significantly larger follicle diameters compared to controls on Day 6 (275.20 ± 12.54 μm; p = 0.037) and Day 8 (277.47 ± 11.47 μm; p = 0.048), although this effect was not maintained by Day 10.

⇒Thank you for your suggestion. We have revised the relevant section as follows:

Follicles treated with DCI exhibited significantly larger diameters than controls on Day 6 (275.20 ± 12.54 μm; p = 0.037) and Day 8 (277.47 ± 11.47 μm; p = 0.048), although this effect was modest and not maintained by Day 10. (Lines 22-24)

Lines 25-27, “The rate of follicular cavity formation was significantly higher in the d-chiro-inositol-treated group on day 6 (p<0.05); however, no significant differences were observed on days 8 and 10.”

“day 6” → should be capitalized consistently (Day 6).

“d-chiro-inositol-treated” → should be abbreviated consistently to DCI-treated.

The correct sentence could be: The rate of follicular cavity formation was significantly higher in the DCI-treated group on Day 6 (p < 0.05); however, no significant differences were observed on Days 8 and 10.

⇒Thank you for your suggestion. We have revised the relevant section as follows:

The rate of follicular antrum formation was significantly higher in the DCI-treated group on Day 6 (p < 0.05); however, no significant differences were observed on Days 8 and 10. (Lines 22-24)

Lines 27-29, In contrast, myo-inositol treatment did not produce significant differences in follicular survival rate, diameter, or cavity formation compared with the control.

Again, use the abbreviation MI for consistency.

“produce significant differences”, better phrasing: “did not affect.”

The correct sentence could be: In contrast, MI treatment did not affect follicular survival, diameter, or cavity formation compared with controls.

⇒Thank you for your suggestion. We have revised the relevant section as you requested.

(Lines 26-28)

Lines 29-31, Estradiol concentrations and relative expression of follicle-stimulating hormone receptor and aromatase genes did not differ significantly between the treatment groups.

“between the treatment groups” could be simplified to “among groups.”

Long phrase could be smoothed.

The correct sentence could be: Estradiol concentrations and expression levels of follicle-stimulating hormone receptor and aromatase genes did not differ significantly among groups.

⇒Thank you for your suggestion. We have revised the relevant section as you requested. (Lines 28-29)

Lines 31-33, Our findings indicate that d-chiro-inositol promotes the transition of preantral to early antral follicles. The decreased myo-inositol/d-chiro-inositol ratio observed in polycystic ovary syndrome may facilitate early follicular development.

“Our findings indicate”, better as “These findings suggest” (more cautious wording for science).

Consistency: use MI/DCI abbreviations instead of repeating full terms.

The correct sentence could be: These findings suggest that DCI promotes the transition from preantral to early antral follicles. The decreased MI/DCI ratio observed in PCOS may facilitate early follicular development.

⇒Thank you for your suggestion. We have revised the relevant section as you requested. (Lines 29-32)

Lines 34-35, Further research is needed to clarify its potential role in clinical applications for polycystic ovary syndrome treatment.

“its potential role”, unclear pronoun (refers to DCI or ratio?).

“clinical applications for PCOS treatment”, better phrasing possible.

The correct sentence could be: Further studies are required to clarify the potential clinical relevance of these findings for PCOS treatment.

⇒Thank you for your suggestion. We have revised the relevant section as you requested. (Lines 32-33)

Introduction:

Line 41, “proportion” is singular; so, the verb should be “manifests”.

⇒Thank you for your suggestion. We have revised the relevant section as you requested. (Line 39)

Line 42, considered to be two-fold, “two-fold” is acceptable, but in scientific writing, “twofold” (without hyphen) is preferred.

The correct form is: “considered twofold”.

⇒Thank you for your suggestion. We have revised the relevant section as you requested. (Line 40)

Line 44-46, "in theca cells (and follicle-stimulating hormone (FSH) receptor and insulin-like growth factor (IGF)-1 receptor expression in granulosa cells)"

The problem is that the nested parentheses are confusing.

The correct form could be: "in theca cells and increase the expression of follicle-stimulating hormone (FSH) and insulin-like growth factor 1 (IGF-1) receptors in granulosa cells"

⇒Thank you for your suggestion. The wording in the relevant section was unclear, so I have revised it as follows.

Elevated luteinizing hormone (LH) levels induce androgen production in theca cells and androgen accelerates development from secondary follicles to small antral follicles. However, a high LH environment inhibits follicle-stimulating hormone (FSH)-dependent follicle development by suppressing FSH receptor expression [3]. Even if LH stimulation causes secondary follicles to grow into antral follicles, these antral follicles may exhibit reduced responsiveness to FSH. (Lines 42-47)

Line 51-52, "Although the pathophysiology of PCOS is complex and not yet fully understood, insulin resistance is believed to be the underlying cause."

The correct or smother form could be: "The pathophysiology of PCOS is complex and not fully understood; however, insulin resistance is believed to be a major contributing factor."

⇒Thank you for your suggestion. We have revised the relevant section as you requested. (lines 49-50)

Line 63-66, "This is because insulin resistance causes low epimerase activity in normal tissues, resulting in systemic DCI deficiency, whereas in the ovaries, insulin sensitivity is preserved, and hyperinsulinemia increases epimerase activity."

This sentence could be split into two sentences to improve clarity.

The correct or smother form could be: "Insulin resistance causes low epimerase activity in normal tissues, resulting in systemic DCI deficiency. In contrast, insulin sensitivity in the ovaries is preserved, and hyperinsulinemia increases epimerase activity."

⇒Thank you for your suggestion. We have revised the relevant section as you requested. (Lines 61-64)

Line 78-80,"Although the efficacy of MI and DCI in the treatment of PCOS has been previously reported, most studies have focused on their metabolic effects, and the direct effects of MI/DCI on follicular development remain insufficiently understood."

The correct form could be: "Although MI and DCI have shown efficacy in treating PCOS, most studies have focused on their metabolic effects. The direct impact of MI/DCI on follicular development remains poorly understood."

⇒Thank you for your suggestion. We have revised the relevant section as you requested. (Lines 75-77)

Some more minor suggestions:

- Avoid repeating “in patients with PCOS” multiple times; vary phrasing.

- Avoid using “this study focused on” repeatedly; consider synonyms like “we investigated” or “we examined”.

- For conciseness, consider combining shorter sentences with semicolons or by rephrasing.

⇒Thank you for this valuable suggestion. We have revised the manuscript to improve clarity and readability by reducing repetitive wording and enhancing sentence structure. Specifically, we replaced repeated occurrences of “in patients with PCOS” with alternative expressions (e.g., “in women with PCOS,” “in the context of PCOS,” or removed the phrase where context made it redundant). We also substituted repeated instances of “this study focused on” with “we investigated,” “we examined,” or “we evaluated.” Additionally, several short or fragmented sentences were combined or restructured to improve conciseness and narrative flow.

Materials and methods:

Some questions:

1- How many animals were used in total across all experiments (not just per group)?

⇒A total of **16 female ICR mice** were used in this study. Added explicit sample count. *Methods*, “Animal and Cell Culture” (Lines 98, 115, 122)

2- Were animals randomized to groups before follicle collection, or were follicles pooled?

⇒Follicles from each mouse were randomly assigned to all groups. To avoid bias, follicles were randomly assigned from the two ovaries of a single mouse. Statement added. (Lines 165-166)

3- Were both ovaries from each mouse used, and if so, were they treated as independent samples or paired?

⇒Yes. Both ovaries from each mouse were used. The left and right ovaries were not distinguished and were used as follicles obtained from a single individual.

4- Was the culture medium serum-containing (5% FBS) throughout, or was serum-free medium also tested?

⇒All cultures were performed using 5% FBS throughout. No serum-free condition was tested.

5- How was the vehicle control for DMSO matched to experimental groups (same final concentration in all groups)?

⇒We added the vehicle such that the DMSO concentration was matched across all groups.

6- Was osmolarity or pH of the culture medium checked after MI/DCI supplementation?

⇒Although we did not measure it, we believe there was no significant change in pH because the culture medium contained phenol red.

7- How was the culture medium collected for E2 measurement-pooled from wells or from individual follicles?

⇒Culture medium was collected individually from each well, not pooled.

8- Were E2 values normalized to follicle number per well?

⇒E2 concentrations were measured per individual follicle, and therefore normalization to follicle count per well was not required.

9- Was a power calculation done to justify the sample size (n = 8 mice)?

⇒In animal experiments, reducing the number of animals is crucial. Based on prior studies, 3–5 animals per experiment were sufficient to detect meaningful effects.

10- Were the data tested for normality before applying ANOVA?

⇒Data distribution was assessed for normality **prior to ANOVA**, using Shapiro–Wilk tests.

11- Were multiple comparisons corrected for (beyond Student-Newman-Keuls)?

⇒Multiple comparisons were adjusted using only the Student-Newman-Keuls method.

Lines 95-97, "The method of euthanasia followed handling was performed in accordance with the guidelines…"

Problem: Unclear and ungrammatical.

The correct form could be: "Euthanasia and subsequent handling were performed in accordance with the guidelines of Sapporo Medical University and the Center for Animal Protection Scientists."

⇒Thank you for your suggestion. We have revised the relevant section as you requested. (Lines 94-96)

Lines 99-100, "sacrificed through intraperitoneal administration of pentobarbital"

Comment: sacrificed is less precise and discouraged. Euthanized is preferred.

The correct form could be: "euthanized via intraperitoneal administration of pentobarbital (120 mg/kg)"

⇒Thank you for your suggestion. We have revised the relevant section as you requested. (Line 98)

Lines 112-114, "…the culture medium was refreshed… and the culture was continued for a total …"

The correct form could be: "The culture medium was refreshed every other day by replacing half of the volume with fresh medium, and culture was continued for a total of 10 days."

⇒Thank you for your suggestion. We have revised the relevant section as you requested. (Lines 111-112)

Lines 133-134, "when the granulosa cells appeared dissolved black"

The problem is using awkward/unclear phrasing.

The correct form could be: "when the granulosa cells appeared degenerated or darkened"

⇒Thank you for your suggestion. We have revised the relevant section as you requested. (Lines 135-136)

Line 139, "E2 concentrations in the culture medium were measured on days…"

The correct form could be: "Measurement of estradiol (E2) concentrations in the culture medium was performed on days 5 and 9 using an ELISA kit (R&D Systems, Minneapolis, MN, USA; detection range: ~0–3.0 ng/mL)."

⇒Thank you for your suggestion. We have revised the relevant section as you requested. (Lines 141-143)

Also, there are some inconsistent units formatting (μM vs uM, spacing in “p<0.001” vs “p < 0.001”), inconsistent gene names (italicize Fshr, Cyp19a1), and missing italics for species (Mus musculus).

⇒Thank you for pointing that out. We have made the correction as you requested.

Results:

Question:

For follicle diameters: are you reporting mean ± SE consistently, or sometimes using SD? The text says SE, but the numbers look like SD ranges.

⇒It is a Standard error.

Comments:

Lines 170-172, that” refers to something singular; follicle diameter is plural.

The correct form could be: “were significantly larger than those in the CTRL group”

⇒Thank you for your suggestion. We have revised the relevant section as you requested. (Line 186)

Lines 216-217, “The E2 concentration in the culture medium on day 5 of the DCI experiment in CTRL, DCI 20 μM, and DCI 50 μM groups were …”

Correction: “…was …”

⇒Thank you for your suggestion. We have revised the relevant section as you requested. (line 228)

Discussion:

Lines 266-267, we found that the addition of DCI promoted the development of secondary follicles from early antral follicles.”

Problem: The phrasing suggests secondary follicles come from antral follicles (biologically confusing).

Correction: “…we found that the addition of DCI promoted the transition of secondary follicles to the early antral stage.”

⇒Thank you for your suggestion. We have revised the relevant section as you requested. (Lines 269-270)

Lines 268-269, “This implies that a reduction in the MI/DCI ratio is closely related to the etiology of PCOS.”

Problem: Too strong for in vitro evidence.

Correction: “…This suggests that a reduction in the MI/DCI ratio may contribute to the pathophysiology of PCOS.”

Lines 273-275, “…follicles cultured without DCI, which were initially delayed in development, eventually caught up with those cultured in the presence of DCI.”

Correction: “…Follicles cultured without DCI, although initially delayed, eventually reached similar developmental stages as those exposed to DCI.”

Lines 310-313, “…humans are monovulatory animals, whereas mice are polyovulatory animals that develop several follicles in one cycle. Therefore, it is normal for murine ovaries to show a multifollicular morphology, and do not fully mimic…”

Correction: “…Therefore, murine ovaries normally show multifollicular morphology, which does not fully mimic human follicle development, especially in PCOS.”

⇒We thank the reviewer for the constructive feedback. To improve clarity, coherence, and accuracy of interpretation, we substantially revised and reorganized the Discussion rather than editing individual sentences. The revised version now more clearly distinguishes the experimental findings, their biological implications, and the limitations of the model.

2. Figures

Figure legends are descriptive but could be streamlined. Ensure all abbreviations are defined at first use in each figure.

⇒We appreciate this practical suggestion. All figure legends have been carefully revised to remove redundancy and ensure that each abbreviation (e.g., DCI, MI, E2, FSHR, Cyp19a1) is defined at its first occurrence. The legends were also streamlined for clarity without loss of essential information.

**Modification:**

Revised *Figure Legends* for Figures 1–5 (Lines **197-218, 234-238, 257-266**).

Example: **Fig 1. Effects of inositol on follicle survival rates**.

Abbreviations: CTRL (control), DCI (D-chiro-inositol), MI (myo-inositol), DMSO (dimethyl sulfoxide).

(a) Survival on Day 10 for CTRL, DCI 20 μM, and DCI 50 μM. The CTRL group received base medium with vehicle (DMSO); DCI groups received base medium with 20 or 50 μM DCI plus DMSO.

(b) Survival on Day 10 for CTRL, MI 20 μM, and MI 50 μM (base medium with MI 20 or 50 μM plus DMSO).

Data are presented as mean ± standard error. One-way ANOVA: no significant differences (panel a: p = 0.161; panel b: p = 0.700).

3. References

⇒We thank the reviewer for this constructive advice. We have added additional references to representative in vitro follicle culture studies beyond our own group to provide a broader methodological context, including works by Hardy et al. 2017 and Telfer et al. (2008). This enhances the connection between our single-follicle model and established culture methodologies.

14.	Telfer EE, Zelinski MB. Ovarian follicle culture: advances and challenges for human and nonhuman primates. Fertil Steril. 2013;99: 1523-1533. doi: [10.1016/j.fertnstert.2013.03.043](https://doi.org/10.1016/j.fertnstert.2013.03.043).

15.	Akiko H, Nahoko M. In vitro culture methods for immature ovarian follicles. J Mamm Ova Res. 2009: 195-201.

Recent reviews/meta-analyses on inositol and PCOS 2023-2024 are cited, which is good. However, authors might expand citations regarding in vitro follicle culture systems beyond their own group’s work.

**Modification:**

Added to *Reference list*. In: Woodruff and shea labs. Follicle handbook:
13; 2014.

Recommendation

Given the issues above, I recommend a Major Revision.

The experimental work is solid and interesting, but the interpretation, discussion depth, and contextualization of findings need significant strengthening.

Language and presentation also need editing before acceptance.

⇒I sincerely appreciate your thorough review. Following your suggestions, I have substantially revised the “Discussion” section to better contextualize the research findings. I have also carefully edited the manuscript to ensure clearer and more consistent expression. I am deeply grateful for your valuable guidance in enhancing the quality of this paper.

Response to Reviewer #2

**Comment 1 — Grammar / Sentence Clarity**

The statement "d-chiro-inositol treatment exhibited significantly larger diameters" is grammatically awkward; it should be rephrased to "follicles treated with d-chiro-inositol exhibited significantly larger diameters..."

**⇒**We thank the reviewer for this helpful linguistic correction. The suggested revision has been applied throughout the *Abstract* and *Results* sections for clarity and grammatical correctness.

**Modification:**

Revised sentences (Lines **22-24**):

“Follicles treated with DCI exhibited significantly larger diameters than controls on Day 6…”

The p-values for the diameter on Day 6 (p=0.037) and Day 8 (p=0.048) are very close to the significance threshold of 0.05, indicating a marginal effect.

**⇒**We appreciate this observation. We have revised the *Results* section to explicitly note that these findings indicate a modest or borderline effect, and should be interpreted cautiously.

**Modification:**

Added clarification (Lines **194-195**):

“However, since the p-value was close to 0.05, the increase in follicle diameter after DCI treatment must be considered a modest effect rather than a robust one.”

The field for keywords is blank in the provided document. This is a major omission.

**⇒**We thank the reviewer for noticing this oversight. We have added appropriate keywords reflecting the scope and focus of this study.

**Modification:**

Added keywords (Lines 319-321):

D-chiro-inositol; myo-inositol; folliculogenesis; secondary follicle; single-follicle culture; PCOS; mouse

The literature review could more sharply define the specific knowledge gap this study aims to fill. It could be more explicit about the lack of in vitro studies isolating the effects of these inositols on specific stages of folliculogenesis.

**⇒**We fully agree. The *Introduction* was revised to clearly state that while many studies examined systemic metabolic effects of inositols in PCOS, few have directly evaluated their local, stage-specific effects on follicular development using isolated culture systems. This addition clarifies the scientific gap addressed in our work.

**Modification:**

Added in *Introduction* (Lines **75-83**):

“Although MI and DCI have shown efficacy in treating PCOS, most studies have focused on their metabolic effects, and the direct impact of MI/DCI on follicular development remains poorly understood. In particular, the influence of altered MI/DCI ratios in PCOS on follicular development has not been fully investigated, and animal model studies are limited. Therefore, this study aimed to examine the direct effects of MI and DCI on secondary follicle development in mice to enhance understanding of PCOS pathophysiology and inform therapeutic strategies. To isolate these stage-specific actions from systemic factors, we employed a single-follicle culture system based on established methods for multistage in vitro follicular development [13–15].”

The text referencing Unfer et al. [14] contains a formatting error with plus signs ("3.00 + 1.22 μM") that should be "±".

**⇒**Thank you for pointing out this formatting issue. The plus signs have been corrected to the standard “±” notation in the *Materials and Methods* section.

**Modification:**

Corrected expression (Lines **128-131**):

“In the same report, MI concentrations in the oocyte fluid were 3.00 ± 1.22 μM for PCOS patients and 22.68 ± 1.38 μM for healthy women; p < 0.001). The DCI concentration in the follicular fluid was 16.65 ± 2.01 μM for PCOS patients and 0.22 +± 0.08 μM for healthy women; p < 0.001.”

The description of the euthanasia method is slightly repetitive and contains a grammatical error in the sentence:

“The method of euthanasia followed handling was performed in accordance with...”

This should be corrected for clarity.

**⇒**We thank the reviewer for noticing this redundancy and grammatical error. The sentence has been rewritten for conciseness and clarity.

**Modification:**

Revised in *Materials and Methods* (Lines **94-98**):

“Euthanasia and subsequent handling were performed in accordance with the guidelines of Sapporo Medical University and the Center for Animal Protection Scientists. The study protocol was approved by the Institutional Animal Care and Use Committee (approval number: 22-051). Six-week-old female ICR mice (n = 16) were euthanized via intraperitoneal administration of pentobarbital (120 mg/kg).”

The use of a high, maximally effective FSH concentration likely masked potential subtle effects of MI.

⇒We agree with this observation and have incorporated it into the *Discussion*. We now explicitly state that the high FSH concentration (approximately 66–67 mIU/mL) may have obscured subtle MI-dependent effects on follicle growth.

**Modification:**

Revised in *Discussion* (Lines **295-296**):

“The high FSH concentration used (~66–67 mIU/mL) may have masked subtle MI-dependent effects.”

The y-axis label in Figure 5, "Change relative to CTRL (log2 transformed)," is ambiguous. It should specify that it represents the log2 of the fold-change.

**⇒**We thank the reviewer for this clear suggestion. The y-axis label in Figure 5 has been revised to read “Log2(fold change relative to control)” for accuracy and clarity.

**Modification:**

Corrected in *Figure 5 Legend and Axis* (Line **259-260**).

Updated label: “log2(fold-change vs CTRL)”.

The section on the mechanism is speculative, as the study did not include experiments to test the proposed PI3K/AKT pathway hypothesis. This should be framed more explicitly as a hypothesis for future research, not a finding.

**Response:**

We fully agree. The relevant sentence in the *Discussion* has been revised to clearly indicate that PI3K/AKT activation is presented as a testable hypothesis rather than a confirmed finding. We also added that future mechanistic studies are warranted.

**Modification:**

Revised in *Discussion* (Lines **290-293**):

“The phosphatidylinositol-3-kinase (PI3K)/AKT signaling pathway, which is known to contribute to follicle activation, may also mediate the effects of DCI [18,19]. This remains a testable hypothesis for future investigation.”

The conclusion could be strengthened by more directly stating that the study provides in vitro evidence for a specific, stage-dependent role of DCI, rather than a general "promotion" of development.

**⇒**We appreciate this constructive feedback. We have revised the *Conclusion* to explicitly state that our findings provide stage-specific, in vitro mechanistic evidence for DCI’s role in early follicle development. We also performed comprehensive proofreading of the entire manuscript for grammar, flow, and readability.

**Modification:**

Revised *Conclusion* (Lines **277-278**):

“However, our findings provide stage-specific, in vitro mechanistic evidence for DCI’s role in promoting the transition from secondary to early antral follicles.”

The manuscript requires careful proofreading for several grammatical errors and awkward phrasing.

⇒Thank you for pointing that out. We performed comprehensive proofreading of the entire manuscript for grammar, flow, and readability.

The flow in some parts of the Methods and Discussion could be improved with minor editorial revisions.

⇒Thank you for pointing this out. We have revised the Methods and Discussion to improve clarity and flow, and we appreciate your helpful feedback.
