# Supplementary material for: Myo-inositol versus D-chiro-inositol in murine in vitro follicular development: An experimental study relevant to polycystic ovary syndrome
Source: PLoS One. 2026 Mar 31;21(3):e0346159. doi: 10.1371/journal.pone.0346159 (PMC13037987; doi:10.1371/journal.pone.0346159)
Supplement: Attachment — Submitted filename: Response_to_Reviewers_auresp_2.docx [file pone.0346159.s003.docx]

We sincerely thank the reviewers for their thorough and constructive evaluation of our manuscript entitled **“Myo-inositol versus D-chiro-inositol in murine in vitro follicular development: an experimental study relevant to polycystic ovary syndrome.”** We have carefully addressed all the comments and revised the manuscript accordingly and performed additional proofreading and minor editorial improvements throughout.

Reviewer #3: Myo-inositol versus D-chiro-inositol in murine in vitro follicular development: an experimental study relevant to polycystic ovary syndrome by Korai et al.

The text has been significantly improved, even if two main concerns remain. First, the little sample size (but probably acceptable for an experimental study). Second, the impossibility to generalize the finding to patients with PCOS. The two points should be more underlined.

⇒Thank you for highlighting these critical limitations. We have strengthened the manuscript by explicitly emphasizing (i) the modest experimental sample size and potential limitations in statistical power, and (ii) the inability to directly generalize findings from healthy murine follicles cultured in vitro to women with PCOS. We also clarified that the present work provides mechanistic, stage-specific in vitro evidence rather than clinical conclusions.

Added in *Abstract* (lines 31-34 in the revised manuscript clean)

“Given the small experimental sample size, the use of healthy murine follicles cultured under a high FSH concentration, and the absence of a PCOS-like ovarian milieu, these findings should be interpreted cautiously and cannot be directly generalized to infertility treatment in women with PCOS.”

Added in *Discussion* (lines 332-338)

“Several limitations should be emphasized. First, the single-follicle murine model has inherent constraints regarding direct clinical extrapolation: mice are polyovulatory and exhibit multifollicular ovarian morphology, whereas humans are monovulatory, and murine follicles have shorter growth periods and distinct endocrine sensitivities compared with human follicles. Second, the experimental sample size was small and multiple follicles were obtained per animal; while this approach aligns with the 3Rs principle, it may limit power and raises the possibility of non-independence among follicles derived from the same mouse.”

Added in *Discussion* (lines 346-351)

“In conclusion, our single-follicle culture experiments provide in vitro evidence for a modest, stage-dependent effect of DCI on early folliculogenesis, characterized by facilitation of the secondary-to-early antral transition, while MI showed no detectable effect under the present high-FSH conditions. These findings are mechanistic and should not be extrapolated to clinical efficacy in PCOS without validation in PCOS-relevant animal models and human follicle systems.”

In addition, the focus of the paper is the PCOS. However, the authors detailed the experimental procedure on mice. It should be interesting to study the effect of inositols on animal model of PCOS.

⇒We acknowledge this important point. We have clarified in the **Discussion** that. We have explicitly stated that future research should test MI/DCI effects in established PCOS animal models (e.g., androgen- or letrozole-induced models) and, where feasible, in human follicle culture systems to evaluate translational relevance.

Added in *Discussion* (lines 300-303)

“Future studies should directly test MI and DCI in established animal models of PCOS (e.g., androgen- or letrozole-induced models) and, where feasible, in human follicle culture systems to evaluate whether similar stage-dependent effects occur in a PCOS-relevant context.”

In the text (see for example the abstract section), the statement “Follicles treated with DCI exhibited.” is confusing.

⇒We revised the abstract for clarity and grammatical correctness to explicitly state the subject and treatment condition (“Follicles treated with DCI exhibited…”), and we restructured the abstract to avoid ambiguous phrasing.

Revised in *Abstract* (lines 22-24)

“Follicles treated with DCI exhibited larger diameters than controls on Day 6 (275.20 ± 12.54 μm; p = 0.037) and Day 8 (277.47 ± 11.47 μm; p = 0.048), indicating a modest, marginally significant effect that was not maintained by Day 10.”

The authors statement “These observations are similar to the decreased MI/DCI ratio observed in PCOS, which may facilitate early follicular development.” should be avoided in the abstract because it has no sense, and in the text should be better supported.

⇒We agree and removed the problematic inference from the abstract. In the main text, we now avoid causal language linking our in vitro results to PCOS pathophysiology and instead frame the relationship as a hypothesis requiring validation in PCOS-relevant models.

Revised in *Abstract* (lines 29-34)

“Together, these data provide in vitro evidence that DCI can facilitate the transition from the secondary (preantral) to the early antral stage under these culture conditions. Given the small experimental sample size, the use of healthy murine follicles cultured under a high FSH concentration, and the absence of a PCOS-like ovarian milieu, these findings should be interpreted cautiously and cannot be directly generalized to infertility treatment in women with PCOS.”

Revised in *Discussion* (lines 297-300)

“we cannot infer that a lower MI/DCI ratio is causally responsible for PCOS follicular dynamics. Accordingly, these findings should be interpreted as mechanistic, stage-specific in vitro observations rather than clinical conclusions.”

Similarly, the conclusion on the PCOS has again no sense.

⇒We revised the conclusion to avoid overgeneralization to PCOS and to state the key finding more precisely as stage-dependent in vitro evidence for DCI under defined culture conditions.

Revised in *Conclusion* (lines 349-351)

“These findings are mechanistic and should not be extrapolated to clinical efficacy in PCOS without validation in PCOS-relevant animal models and human follicle systems.”

The introduction is focused on PCOS. The mechanism of the anovulation in PCOS should be referenced and, probably, is more complex of those reported.

⇒Thank you for your suggestion. I agree that anovulation in PCOS is multifactorial. I have revised the introduction with appropriate references as follows.

Revised in *Introduction* (lines 54-64)

“Mechanistically, persistently rapid GnRH pulsatility in PCOS has been suggested to favor LH hypersecretion and an elevated LH/FSH ratio, potentially creating an LH-dominant endocrine milieu in which the FSH-dependent granulosa cell differentiation required for dominant follicle selection and preovulatory maturation may be less likely to proceed normally [3-5]. In addition, anti-Müllerian hormone (AMH), often elevated in PCOS due to an increased pool of small antral follicles, has been proposed to attenuate follicular sensitivity to FSH and to limit aromatase induction, which could contribute to impaired follicle selection and maturation [7,8]. Taken together, ovulatory failure in PCOS may be viewed as an expansion of the small antral follicle cohort accompanied by reduced FSH responsiveness and suboptimal selection/maturation, culminating in anovulation and polycystic ovarian morphology [3,7,8].”

Data on inositols reported in the introduction are not balanced. The authors should underline that inositols have no evidence-based role for the treatment of infertility in PCOS (see new guidelines on PCOS).

⇒We revised the introduction to present a balanced view. We now explicitly acknowledge that evidence for reproductive endpoints is limited and that recent international evidence-based PCOS guidelines do not consider inositol an evidence-based first-line therapy for infertility in PCOS. The rationale for this study is mechanistic rather than therapeutic.

Revised in Introduction (lines 84-93)

“Clinical data on inositol supplementation in PCOS are mixed, with heterogeneity in formulations, dosing, and outcomes, and the overall certainty of evidence for reproductive endpoints remains limited. Recent international evidence-based PCOS guidelines emphasize that metformin has greater efficacy for metabolic features than inositol and that the clinical benefits of inositol are limited; therefore, inositol is not considered an evidence-based, first-line therapy for infertility in PCOS and, at most, may be considered via shared decision-making in selected individuals [15,16]. Nevertheless, both MI and DCI have been investigated for potential effects on metabolic and hormonal parameters in PCOS, and combination regimens (e.g., MI:DCI 40:1) have been proposed, although definitive evidence for improved fertility outcomes remains lacking.”

The authors stated that “In healthy individuals, the MI/DCI ratio in the follicular fluid is 100:1”. This means that the “normal” ovary needs of high concentrations of myo-inositol. How can the authors explain this discrepancy?

⇒Thank you for clarifying this point. We agree that the physiological MI enrichment of follicular fluid does not necessarily predict that additional MI supplementation will further enhance follicle growth in every in vitro setting. We believe that apparent “discrepancy” can be explained by three, non-mutually exclusive reasons:

1, Requirement vs. supplementation (baseline sufficiency):

A high MI/DCI ratio in vivo is consistent with MI being a baseline substrate that the follicle normally maintains at sufficient levels. In our system, follicles were cultured in standard media that already contain inositol and other nutrients, and follicles may also retain endogenous MI stores. Under such “MI-sufficient” conditions, adding extra MI may not produce a measurable incremental effect.

2, Ceiling/saturation effects due to high FSH conditions:

We intentionally used a high, maximally effective FSH concentration to ensure robust growth across groups. Under near-saturating gonadotropin stimulation, subtle modulatory effects of MI (e.g., on FSH signaling efficiency or metabolism) could be masked, making it difficult to detect an additional benefit of MI supplementation.

3, Model context and stage specificity:

MI’s clinical and physiological relevance in PCOS has often been discussed in relation to metabolic/insulin-related pathways and later follicular selection/maturation in a PCOS-like milieu. Our model used healthy murine follicles and did not recreate the endocrine–metabolic environment of PCOS, nor did it fully model later-stage follicular arrest. Therefore, the absence of a significant MI “add-on” effect in our defined, MI-sufficient, high-FSH culture does not contradict the concept that MI is physiologically important in vivo; rather, it indicates that MI supplementation may have limited incremental impact under these specific experimental conditions.

We revised the manuscript to explicitly distinguish “physiological enrichment/requirement” from “supplementation benefit,” and we added this as a key limitation and future direction (e.g., testing MI dose–response at lower FSH and in PCOS animal models).

Revised in *Discussion* (lines 314-331)

“MI, known as a second messenger of FSH, did not significantly affect follicle growth or E2 levels in this study. Importantly, the physiological enrichment of MI in follicular fluid does not necessarily imply that additional MI supplementation will further enhance follicle development under all in vitro conditions. One explanation is that our culture system was likely MI-sufficient at baseline (e.g., standard media components and/or endogenous MI retained within follicles), such that extra MI provided limited incremental benefit. In addition, the high, near-maximally effective FSH concentration used (~66–67 mIU/mL) may have introduced a ceiling effect, masking subtle MI-dependent modulation of FSH responsiveness or cellular metabolism. Moreover, the MI concentrations applied (20–50 μM) were selected to reflect physiological or PCOS follicular-fluid levels in vivo, whereas many in vitro maturation studies use millimolar concentrations [24]. Future studies should therefore evaluate MI in a dose–response manner under conditions with greater dynamic range (e.g., lower FSH stimulation and/or defined MI-depleted media, with careful control of osmolarity) and, to improve translational relevance, in PCOS-relevant endocrine–metabolic contexts. Taken together, the lack of an MI “add-on” effect in our MI-sufficient, high-FSH culture system should not be interpreted as evidence against MI’s physiological importance in vivo, nor does it support clinical efficacy in PCOS without validation in PCOS-relevant models and human systems.”

Added in *Discussion* (lines 339-343)

“Third, the high, maximally effective FSH concentration used to support robust growth may have reduced sensitivity to detect subtle MI-dependent effects or smaller treatment differences. Fourth, only two concentrations of each inositol were tested, and MI:DCI combination ratios, long-term oocyte competence endpoints, or downstream signaling readouts were not evaluated.”

The discussion is short and superficial. The study limitations are totally lacking as well as the future researches.

⇒We substantially expanded the Discussion to include (i) cautious interpretation of marginal effects, (ii) clear limitations, and (iii) concrete future research directions including PCOS animal models, dose-response, lower FSH conditions, and pathway validation.

Added in *Discussion* (lines 287-291)

“In this study, DCI supplementation in a single-follicle murine culture system was associated with a modestly earlier increase in follicle diameter and a higher antrum formation rate on Day 6, suggesting facilitation of the secondary-to-early antral transition. However, the diameter differences were small, only marginally significant at some time points (p-values near 0.05), and not sustained through Day 10.”

Added in *Discussion* (lines 292-303)

“Follicles cultured without DCI initially showed delayed development but ultimately reached the same stage of development as follicles exposed to DCI. Therefore, DCI supplementation in our system did not reproduce the late-stage arrest typical of PCOS. This indicates that the observed effect is stage-limited and context-dependent. Because our experiments used healthy murine follicles and did not recreate the hyperandrogenic, hyperinsulinemic, or inflammatory ovarian milieu characteristic of PCOS, we cannot infer that a lower MI/DCI ratio is causally responsible for PCOS follicular dynamics. Accordingly, these findings should be interpreted as mechanistic, stage-specific in vitro observations rather than clinical conclusions. Future studies should directly test MI and DCI in established animal models of PCOS (e.g., androgen- or letrozole-induced models) and, where feasible, in human follicle culture systems to evaluate whether similar stage-dependent effects occur in a PCOS-relevant context.”

Added in *Discussion* (lines 332-345)

“Several limitations should be emphasized. First, the single-follicle murine model has inherent constraints regarding direct clinical extrapolation: mice are polyovulatory and exhibit multifollicular ovarian morphology, whereas humans are monovulatory, and murine follicles have shorter growth periods and distinct endocrine sensitivities compared with human follicles. Second, the experimental sample size was small and multiple follicles were obtained per animal; while this approach aligns with the 3Rs principle, it may limit power and raises the possibility of non-independence among follicles derived from the same mouse. Third, the high, maximally effective FSH concentration used to support robust growth may have reduced sensitivity to detect subtle MI-dependent effects or smaller treatment differences. Fourth, only two concentrations of each inositol were tested, and MI:DCI combination ratios, long-term oocyte competence endpoints, or downstream signaling readouts were not evaluated. Finally, mechanistic interpretation remains limited because PI3K/AKT (or other pathway) activation was not directly measured; these pathways should be interrogated experimentally in future work.”

Reviewer #4: Myo-inositol versus D-chiro-inositol in murine in vitro follicular development: an experimental study relevant to polycystic ovary syndrome by Korai et al.

The statement "d-chiro-inositol treatment exhibited significantly larger diameters" is grammatically awkward; it should be rephrased to "follicles treated with d-chiro-inositol exhibited significantly larger diameters..."

⇒Thank you for this suggestion. We agree with the reviewer that the phrasing should clearly indicate the experimental unit (follicles). We note that this specific wording appears to reflect an earlier version. In the current revised manuscript, we use “Follicles treated with DCI exhibited significantly larger diameters …” in the Abstract and relevant sections. (lines 22-23)

The p-values for the diameter on Day 6 (p=0.037) and Day 8 (p=0.048) are very close to the significance threshold of 0.05, indicating a marginal effect.

⇒We agree and now explicitly describe these findings as modest and marginal, and we caution against overinterpretation.

Revised in *Abstract* (lines 22-24)

“Follicles treated with DCI exhibited larger diameters than controls on Day 6 (275.20 ± 12.54 μm; p = 0.037) and Day 8 (277.47 ± 11.47 μm; p = 0.048), indicating a modest, marginally significant effect that was not maintained by Day 10.”

The field for keywords is blank in the provided document. This is a major omission.

⇒Thank you for pointing this out. We apologize for any confusion. The Keywords have now been added to the manuscript text under the heading “Keywords” (lines 354-356).

The literature review could more sharply define the specific knowledge gap this study aims to fill. It could be more explicit about the lack of in vitro studies isolating the effects of these inositols on specific stages of folliculogenesis.

⇒Thank you. We revised the Introduction to explicitly state the knowledge gap (few controlled in vitro studies isolating stage-specific actions of MI vs DCI) and clarified how the single-follicle culture system addresses this gap.

Added in *Introduction* (lines 94-102)

“Despite extensive clinical and metabolic research, a key knowledge gap remains: there are few controlled in vitro studies that isolate the direct, stage-specific actions of MI versus DCI on folliculogenesis independent of systemic endocrine and metabolic factors. In particular, it is unclear whether changes in MI/DCI exposure can directly influence the secondary-to-early antral transition, a stage implicated in the early acceleration observed in PCOS. To address this gap, we examined the effects of MI and DCI on isolated murine secondary follicles using a single-follicle culture platform, enabling quantification of survival, growth, antrum formation, steroid output, and selected granulosa-cell transcripts under defined conditions [17–19].”

The text referencing Unfer, et al. [14] contains a formatting error with plus signs ("3.00 + 1.22 μM") that should be "±".

⇒Thank you for the suggestion. We note that this wording reflects an earlier version; in the revised manuscript, we used “3.00 ± 1.22 μM” (line 147).

The description of the euthanasia method is slightly repetitive and contains a grammatical error in the sentence: "The method of euthanasia followed handling was performed in accordance with..." This should be corrected for clarity.

⇒Thank you for the suggestion. The revised paper eliminates redundancy and rewrites sentences for clarity. It explicitly states that euthanasia and subsequent handling were performed in accordance with institutional guidelines. (lines 112-116)

The use of a high, maximally effective FSH concentration likely masked potential subtle effects of MI.

⇒We agree. We strengthened this point in the Discussion, explicitly describing a potential ceiling effect under the high FSH concentration (~66–67 mIU/mL), which could reduce sensitivity to detect MI-dependent modulation.

Added in *Discussion* (lines 319-322)

“In addition, the high, near-maximally effective FSH concentration used (~66–67 mIU/mL) may have introduced a ceiling effect, masking subtle MI-dependent modulation of FSH responsiveness or cellular metabolism.”

The y-axis label in Figure 5, "Change relative to CTRL (log2 transformed)," is ambiguous. It should specify that it represents the log2 of the fold-change.

⇒Thank you for this suggestion. The revised version explicitly states, “Data are presented as log2(fold-change vs CTRL).” (lines 277-278)

The authors acknowledge the limitation that their model did not fully replicate the arrested development seen in later-stage PCOS follicles.

⇒We agree and clarified that DCI supplementation in our system did not reproduce the late-stage arrest typical of PCOS, and therefore findings should be interpreted as stage-specific in vitro observations.

Added in *Discussion* (lines 292-295)

“Follicles cultured without DCI initially showed delayed development but ultimately reached the same stage of development as follicles exposed to DCI. Therefore, DCI supplementation in our system did not reproduce the late-stage arrest typical of PCOS. This indicates that the observed effect is stage-limited and context-dependent.”

The section on the mechanism is speculative, as the study did not include experiments to test the proposed PI3K/AKT pathway hypothesis. This should be framed more explicitly as a hypothesis for future research, not a finding.

⇒We agree and revised the language to clearly indicate that PI3K/AKT involvement is a testable hypothesis, not a demonstrated finding. The revised version adds explicit wording “This remains a testable hypothesis for future investigation.” (lines 312-313)

Added in *Discussion* (lines 343-345)

“Finally, mechanistic interpretation remains limited because PI3K/AKT (or other pathway) activation was not directly measured; these pathways should be interrogated experimentally in future work.”

The conclusion could be strengthened by more directly stating that the study provides in vitro evidence for a specific, stage-dependent role of DCI, rather than a general "promotion" of development.

⇒Thank you for this suggestion. We revised the conclusion to state more directly that the study provides in vitro evidence for a stage-dependent effect of DCI on the secondary-to-early antral transition.

Added in *Discussion* (lines 346-348)

“In conclusion, our single-follicle culture experiments provide in vitro evidence for a modest, stage-dependent effect of DCI on early folliculogenesis, characterized by facilitation of the secondary-to-early antral transition,”

The manuscript requires careful proofreading for several instances of grammatical errors and awkward phrasing.

⇒We agree and performed careful proofreading throughout the manuscript, correcting grammar and improving readability while keeping scientific meaning unchanged.

The flow in some parts of the Methods and Discussion could be improved with minor editorial revisions.

⇒Thank you. We implemented minor editorial revisions to improve logical flow and readability, particularly around study limitations and interpretation.

We believe these revisions significantly strengthen the manuscript’s clarity, rigor, and scientific balance. We are grateful to the Reviewers and Editor for their insightful guidance and hope that the revised version is now suitable for publication.
